# Proviruses with Long-Term Stable Expression Accumulate in Transcriptionally Active Chromatin Close to the Gene Regulatory Elements: Comparison of ASLV-, HIV- and MLV-Derived Vectors

**DOI:** 10.3390/v10030116

**Published:** 2018-03-08

**Authors:** Dalibor Miklík, Filip Šenigl, Jiří Hejnar

**Affiliations:** 1 Institute of Molecular Genetics, Czech Academy of Sciences, Videnska 1083, CZ-14220 Prague 4, Czech Republic; Dalibor.Miklik@img.cas.cz (D.M.); Filip.Senigl@img.cas.cz (F.S.); 2 Faculty of Science, Charles University, Albertov 6, CZ-12843 Prague 2, Czech Republic

**Keywords:** retrovirus integration, provirus silencing, gene regulatory elements, genome-wide provirus distribution

## Abstract

Individual groups of retroviruses and retroviral vectors differ in their integration site preference and interaction with the host genome. Hence, immediately after infection genome-wide distribution of integrated proviruses is non-random. During long-term in vitro or persistent in vivo infection, the genomic position and chromatin environment of the provirus affects its transcriptional activity. Thus, a selection of long-term stably expressed proviruses and elimination of proviruses, which have been gradually silenced by epigenetic mechanisms, helps in the identification of genomic compartments permissive for proviral transcription. We compare here the extent and time course of provirus silencing in single cell clones of the K562 human myeloid lymphoblastoma cell line that have been infected with retroviral reporter vectors derived from avian sarcoma/leukosis virus (ASLV), human immunodeficiency virus type 1 (HIV) and murine leukaemia virus (MLV). While MLV proviruses remain transcriptionally active, ASLV proviruses are prone to rapid silencing. The HIV provirus displays gradual silencing only after an extended time period in culture. The analysis of integration sites of long-term stably expressed proviruses shows a strong bias for some genomic features—especially integration close to the transcription start sites of active transcription units. Furthermore, complex analysis of histone modifications enriched at the site of integration points to the accumulation of proviruses of all three groups in gene regulatory segments, particularly close to the enhancer loci. We conclude that the proximity to active regulatory chromatin segments correlates with stable provirus expression in various retroviral species.

## 1. Introduction

Integration of retroviral genomic DNA into the host genome is a key step in the retroviral replication cycle. Although there are reports regarding the expression of retroviral genes from an unintegrated genome [1], effective and long-term expression occurs only from the integrated form of the retroviral genome—the provirus. However, the provirus expression is highly variable with individual proviruses either epigenetically silenced, fully expressed or alternating between these two states. The silencing of proviral expression poses a drawback for the utilization of retroviruses as vectors where long-term stable expression is the desired outcome. In the clinic, the existence of a cure-resistant reservoir of latent proviruses in HIV-infected patients is the principal obstacle of sterilizing and functional antiretroviral therapy. The question then arises to what level the chromatin surrounding the provirus determines the heterogeneity of proviral expression.

Most retroviruses are known to non-randomly target the host genome. Human immunodeficiency virus type 1 (HIV) and related lentiviruses preferentially integrate into active genes [2,3,4,5,6] whereas murine leukaemia virus (MLV) and other gammaretroviruses prefer to integrate into active transcriptional start sites (TSS) and enhancers [4,5,7]. On the other hand, avian sarcoma and leukosis virus (ASLV) display random-like integration exhibiting a weak preference for active genes [5,8,9].

These integration preferences are driven by cellular targeting factors, which bind to retroviral integrase and have affinity for certain histone marks. In this way, the retroviral intasomes tether to specific chromatin regions that are enriched at respective histone marks. For HIV, two proteins that are critical for the targeting of HIV proviruses have been identified, lens epithelium-derived growth factor (LEDGF/p75) and cleavage and polyadenylation specific factor 6 (CPSF6). LEDGF/p75 binds the integrase of HIV, enhances integration and targets intasomes to active genes [10,11,12] and nuclear periphery [13] while CPSF6 has been shown to interact with the capsid protein of HIV and plays an important role in the targeting of transcribed genes [14,15]. Integrase of MLV interacts with the bromodomain and extraterminal (BET) proteins, which results in MLV integration close to active promoters and enhancers [16,17,18]. Until recently, ASLV was thought to lack an integrase-binding partner important for the integration process. However, a recent study identified the histone chaperone FACT (FAcilitates Chromatin Transcription) complex as a cofactor of ASLV integrase promoting its integration [19].

Disruption of the interaction between the intasome and BET proteins or LEDGF/p75 has resulted in the decreased efficiency of retrovirus integration and retargeting outside the preferred genomic regions [12,20]. Hence, retargeting strategies are already being used to design the next generation of retroviral vectors [20,21,22] that demonstrate the decreased risk of cellular protooncogene transactivation [23]. In addition to genetic manipulation of retroviral integrase, specific HIV-1 integrase-LEDGF/p75 interaction can also be allosterically inhibited with small molecules of LEDGF/p75 inhibitors (LEDGINs), which represents a new and promising therapeutic strategy [24].

Functional studies correlating the integration site environment and provirus transcription have mostly focused on the HIV latent reservoir—a population of proviruses with epigenetically silenced expression in certain cell niches [25]. Early studies suggested the importance of the proper genomic environment for retroviral expression [26,27]. Analysis of latently infected cellular clones suggested gene deserts, centromeric heterochromatin and, surprisingly, highly active genes as target sites that promote provirus silencing [28]. At these target sites, HIV copies are assumed to be more vulnerable to the executive mechanisms of provirus silencing such as DNA methylation and histone modifications [29,30,31]. Nevertheless, the association of genomic features with silenced proviruses is less clear when fractions of latently HIV-infected cells have been studied in primary human T cells or a T cell line [32]. Quite recently, retargeting of HIV integration away from active genes was reported to increase the latent reservoir and reduce the reactivation by latency-reversing agents [33]. The proviral activity and reactivation potential can also correlate with the distance to H3K27 acetylated sites [34], which are markers of active promoters and enhancers.

In parallel to HIV, the link between integration sites and proviral expression has also been studied in ASLV-infected mammalian cells. Non-avian cells are not permissive for ASLV [35,36] and ASLV proviruses are efficiently silenced [37,38]. Therefore, the selection of genomic sites supporting proviral expression is quite stringent. We have shown that the mechanism of ASLV silencing depends on position of the provirus in the genome. When integrated within the body of transcribed genes, proviral expression is silenced in a de novo DNA methyltransferase-dependent manner whereas outside of genes, the silencing is DNA methylation-independent [39]. Proviruses integrated in a close proximity to highly active transcription start sites remain mostly unaffected by silencing [39,40]. ASLV silencing within gene bodies can be overcome by the insertion of CpG-island core elements, which protect proviral long terminal repeats (LTR) from methylation [40,41]. In this case, stably active proviruses were found not only in the proximity to active TSS but also in distal parts of the active gene bodies and close to active enhancers. Our studies have shown that ASLV is a valuable tool for the study of the relationship between integration site and proviral expression, which must be studied in both a genomic and epigenomic context.

In the present study, we compared the patterns of provirus expression in single cell clones infected with retroviral vectors derived from ASLV, HIV and MLV. The analysis of integration sites of stably active proviruses showed that some genomic and epigenomic features, especially proximity to active regulatory segments, correlate with stable provirus expression of various retroviral species.

## 2. Materials and Methods

### 2.1. Construction of the Retroviral Vectors

**Figure 1:**
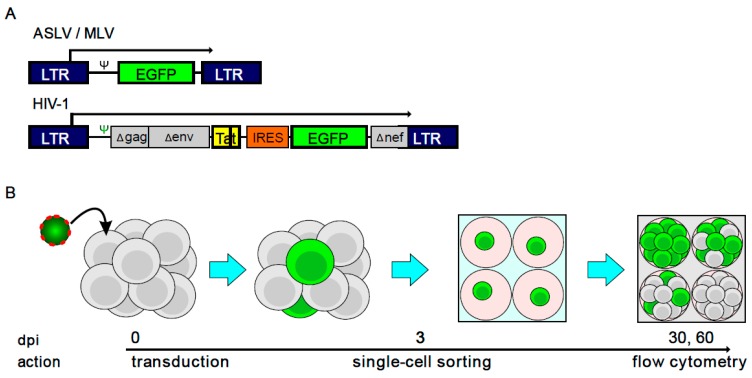
Vectors and the methodological approach used in this study to isolate single-cell clones with stable active proviruses. (**A**) Schematic representation of retroviral vectors. Avian sarcoma/leukosis virus (ASLV)- and murine leukaemia virus (MLV)-based vectors are of the same structure containing just the respective long terminal repeats (LTR) and enhanced green fluorescent protein (EGFP) for proviral activity detection. The human immunodeficiency virus (HIV)-based vector contains deleted fragments of *gag, env* and *nef* genes, full-length tat and EGFP genes and internal ribosomal entry site (IRES). Transcription start sites (TSS) are denoted by broken arrows. ψ, packaging signal. (**B**) The workflow in obtaining the single-cell clones. K562 cell line was transduced with low multiplicity of infection (MOI) of VSV-G-pseudotyped ASLV-, MLV- or HIV-based vectors. Three days post infection (dpi), Green fluorescent protein positive (GFP^+^) cells were single-cell sorted to a 96-well plate and cellular clones were established. Cellular clones were examined for the percentage of GFP^+^ cells 30 and 60 dpi by flow cytometry. Clones containing ≥90% of GFP^+^ cells were subjected to further examination of proviral integration sites.

The construction of plasmids used for the propagation of LTR-driven enhanced green fluorescent protein (EGFP)-expressing vectors (EV731, pLG and pAG3, see Figure 1A) derived from HIV, MLV and ASLV, respectively, was described previously [26,39,42]. HIV-1-derived vector is bicistronic with transactivating Tat gene necessary for the elongation phase of HIV-1 LTR driven transcription, internal ribosomal entry site (IRES) and EGFP (Figure 1A).

### 2.2. Cell Culture and Virus Propagation

Propagation of retroviral vector AG3 using the AviPack packaging cell line was part of the previous study and is described there [39]. MLV-based LG vector was propagated as described previously [42]. Briefly, GP-293 packaging cell line (Clontech, Mountain View, CA, USA) was calcium phosphate co-transfected with 50 μg pLG and 10 μg pVSV-G plasmids (Clontech). HIV-derived vector was produced by HEK293T cell line calcium phosphate co-transfection of 10 µg of pEV731 (LTR-Tat-IRES-EGFP-LTR vector the pHR’ series [26]), 10 µg of psPAX2 (Clontech) and 10 µg of pVSV-G (Clontech). Viral stocks were collected 48 h after transfection, frozen and stored in −80 °C. Titration of the infectious virus stock was performed by serial dilution and subsequent infection of K562 cells. Three days post infection (dpi), the number of GFP-positive (GFP^+^) cells was counted by LSRII flow cytometer (Becton Dickinson, Franklin Lakes, NJ, USA). The GP-293 and HEK293 cell lines were maintained in D-MEM/F12 (Sigma, St. Louis, MO, USA) with 5% new-born calf serum, 5% foetal calf serum (both Gibco BRL, Waltham, MA, USA) and penicillin/streptomycin (100 mg/mL each, Sigma) in a 5% CO_2_ atmosphere at 37 °C. K562 human myeloid lymphoblastoma cell line was maintained in RPMI 1640 supplemented with 5% new-born calf serum, 5% foetal calf serum and penicillin/streptomycin (100 mg/mL each, Sigma) in a 5% CO_2_ atmosphere at 37 °C.

### 2.3. Infection and Subcloning of K562 Cells

The K562 cell line was infected and single-cell sorting was performed according to the previously described protocol [40]. Cells were infected with low multiplicity of infection (MOI) to ensure that the amount of GFP^+^ cells 3 dpi did not exceed 1% of infected cell culture. Three dpi, GFP^+^ cells were sorted in single-cell sort mode using an Influx cell sorter (Becton-Dickinson, Franklin Lakes, NJ, USA) into 96-well tissue culture plates to obtain single-cell clones. Expanded clones were sub-cultured by passaging 25% of the cells three times a week. The percentage of GFP^+^ cells was assessed 30 and 60 dpi with the LSRII cytometer. Clones containing ≥90% GFP^+^ cells in the clonal population 60 dpi were arbitrarily regarded as stably expressing clones. For ASLV and HIV-1 clones, genomic DNA was isolated separately from each clone. In the case of MLV, clones exhibiting stable GFP expression were pooled and collected for DNA isolation.

### 2.4. Cloning and Sequencing of Provirus Integration Sites

The provirus-cell DNA junction sequences were amplified using the splinkerette-PCR method [43]. The protocol for the cloning and sequencing of ASLV integration sites was described previously [40]. Briefly, a splinker adapter was ligated to restriction enzyme-digested genomic DNA of each clone and nested PCR followed by Sanger sequencing was performed. For digestion of genomic DNA, different restriction enzymes compatible with the sequence of proviral DNA were chosen. For HIV, NlaIII, MseI, or a mix of SpeI, NheI and XbaI (SNX mix) was used. NlaIII was used in the first digestion reaction for MLV-transduced clones. After the ligation of splinker adaptors, PvuII (HIV) or ClaI (MLV) was used in the second digestion to prevent possible amplification of inner proviral sequences. For nested PCR, the following LTR-specific primers were used: HIV-1 spLTR: TATCTGATCCCTGGCCCTGGTGTGTAG, HIV-1 spinLTR: CTGCCAATCAGGGAAGTAGCCTTGTGTG, MLV spLTR: TTCCATGCCTTGCAAAATGGCGT, MLV spinLTR: TGGCGTTACTTAAGCTAGCTTGCC.

### 2.5. Mapping and Genomic Characterization of Provirus Integration Sites

All junction sequences containing the end of 5’LTR and the unique cellular DNA sequence obtained from the splinkerette PCR were mapped to the Feb. 2009 human genome assembly (hg19) using BLAT found on the UCSC Genome Browser website (http://genome.ucsc.edu/). Genomic coordinates of the LTR-proximal nucleotide of the obtained genomic sequences with unique score were considered as positions of integration.

### 2.6. Uniquely Mapped Matched Random Controls

To create a set of random genomic positions with position-to-restriction enzyme-recognized site distribution, the restrSiteUtils_1.2.8 R package was used [44]. A set of 200 matched random positions per integration site was generated. Genomic sequences covering ranges from random position to restriction enzyme-recognized site were extracted from hg19 assembly using Biostrings R package [45] for each matched random position. Sequences were mapped to hg19 assembly using BLAT and sequences with single full-length match with ≥98% identity were accepted as uniquely mapped matched random controls (umMRCs). Three umMRCs per integration site were randomly selected and used as controls in subsequent analysis.

### 2.7. Data Source and Integration Site Analysis

Genomic and epigenomic data for in silico analysis of features associated with integration sites and umMRCs were obtained from UCSC golden path (http://hgdownload.cse.ucsc.edu/goldenPath/hg19/database/). The sources are described in more detail in [40]. Data for cap analysis of gene expression (CAGE)-peak positions (FANTOM Consortium and the RIKEN PMI and CLST (DGT)) were obtained from the web-based data source (http://fantom.gsc.riken.jp/5/). Analysis of distances from, and the frequency of, targeting of the features was performed using the GenomicRanges R package [46].

### 2.8. Active Genes

A RefSeq Gene was considered to be active if its TSS was either within the H3K4me3 peak (for the selection of histone modification peaks see [40]), or within the Tss chromatin segment, or within the distance of 500 bp from the nearest CAGE peak TSS (FANTOM Consortium and the RIKEN PMI and CLST (DGT)). Three groups of active genes/TSS were analysed separately.

### 2.9. Merged Chromatin Segments

In order to simplify the analysis associating integrated proviruses to chromatin states, twenty-five chromatin segments that were generated using Hidden Markov Model-based modelling [47] were merged by the itemRGB field to create Active chromatin segments and Regulatory segments. The active chromatin segments group contains 18 segments including Tss, TssF, PromF, PromP, Enh, EnhF, EnhW, EnhWF, DnaseD, DnaseU, FaireW, Gen5, Elon, ElonW, ElonWF, Gen3, H4K20 and Low segments. The regulatory segments group consists of 11 segments including Tss, TssF, PromF, PromP, Enh, EnhF, EnhW, EnhWF, DnaseD, DnaseU and FaireW.

### 2.10. Active Gene Matched Random Controls

One thousand umMRCs per integration site were generated. For each umMRC, the distance to active RefSeq genes whose TSSs associated with a Tss chromatin segment was counted in the same manner as performed for integration site analysis. For each integration site, the three umMRCs with the most similar distance to an active gene when compared to integration site were randomly selected.

### 2.11. Statistics

R software version 3.3.2. was used for statistical analysis. To count the *p*-value of the frequencies of targeting the features Fisher’s Exact Test for Count Data was used. To count the *p*-value of the distances to the features, Wilcoxon signed rank test was used.

## 3. Results

### 3.1. Stability of Proviral Expression

To examine the stability of proviral expression, we used vectors derived from HIV and MLV in a clonal assay, which is schematically depicted in Figure 1B. A miniviral vector transducing EGFP as an expression marker was pseudotyped with VSV-G and used to transduce the human myeloblastoma cell line K562. Three dpi, cells that were positive for GFP expression (GFP^+^) were single-cell sorted and expanded to clonal populations. The established cellular clones were then examined for the number of cells expressing EGFP at 30 and 60 dpi. Clones containing at least 90% GFP^+^ cells 60 dpi were regarded as clones which contain stably active proviruses. For comparison, we included the dataset of K562 clones containing the ASLV-derived pAG vector. These clones were obtained in analogous experiment described by Senigl et al. [40] (pAG3 vector).

**Figure 2:**
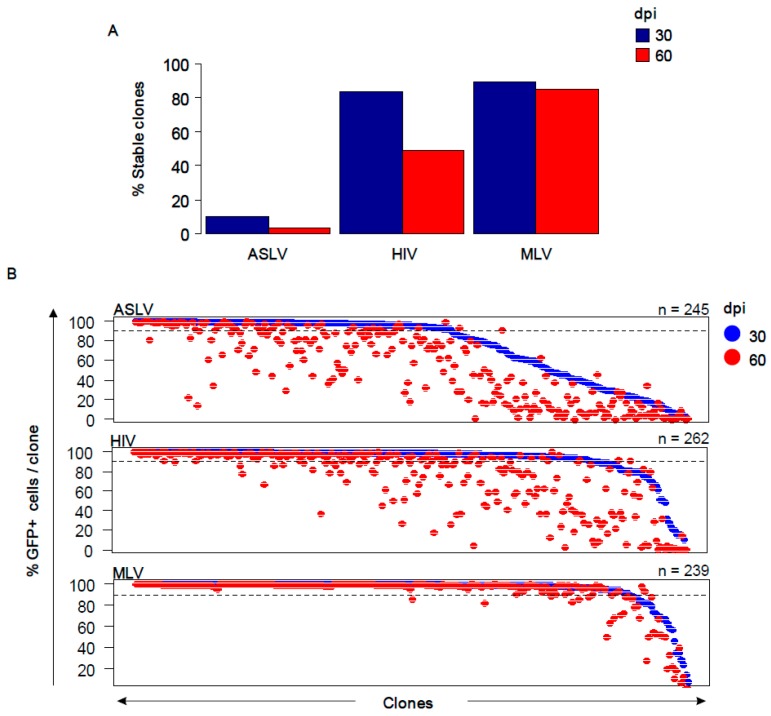
Differential expression stability of vectors. (**A**) The percentage of clones with stable active proviruses (≥90% GFP^+^ cells) at 30 dpi (blue columns) and 60 dpi (red columns). 100% represents the number of clones obtained after single-cell sorting of GFP^+^ cells 3 dpi. (**B**) The percentage of GFP^+^ cells in single-cell clones analysed at 30 and 60 dpi. Each clone is represented by one blue (30 dpi) and one red (60 dpi) dot at the same position along the x-axis. In most cases, the red dot is lower along the *y*-axis than the blue dot, which represents the silencing of GFP expression between 30 and 60 dpi. Along the *x*-axis, clones are ordered by the percentage of GFP^+^ cells at 30 dpi. The dashed line marks the value of 90% of GFP^+^ cells. The numbers of clones analysed up to 60 dpi are depicted.

The percentages of GFP^+^ cells were determined in 2128 ASLV, 378 HIV and 239 MLV-transduced cell clones at 30 dpi. ASLV was effectively silenced in the K562 cell line with only 11.5% of the clones (245) maintaining provirus expression. In contrast, more than 80% of HIV- and MLV-transduced clones (262 and 239, respectively) maintained stable expression at 30 dpi (Figure 2A). The clones with stable provirus expression 30 dpi were cultured for additional 30 days and the percentage of GFP^+^ cells was calculated again at 60 dpi. Whereas almost all MLV-transduced clones (202) maintained provirus expression, the numbers of clones with stable ASLV and HIV expression further decreased to 3% (74) and 49% (136), respectively (Figure 2B). Thus, MLV provirus expression was deemed to be long-term stable. HIV provirus expression was observed to be stable during short cultivation (30 dpi) but displayed gradual silencing when cultured for a longer period. We also used the FACS data to assess the GFP fluorescence intensity produced by ASLV, HIV and MLV vectors in clones expanded 30 dpi. HIV proviruses displayed the highest GFP fluorescence intensity, followed by MLV and ASLV (Supplementary Figure S1).

### 3.2. Gene Targeting and Distance to TSS

Cell clones infected with ASLV-, HIV- and MLV-derived vectors containing ≥90% of GFP^+^ cells at 60 dpi were subjected to splinkerette PCR in order to amplify and sequence the provirus integration sites. ASLV- and HIV-transduced clones were analysed individually, whereas the MLV-transduced clones were pooled and analysed en masse, because these clones are uniform as to the provirus stability (Figure 2B) and their individual analysis does not provide any additional information. We identified 45 HIV and 32 MLV integration sites, which are together with 46 ASLV integration sites given in Supplementary Table S1. To each set of integration sites, uniquely mapped matched random controls (umMRC) were generated. These controls exhibit the same distribution of distances from the recognition sites of a given restriction enzyme and the sequences of the control site fulfilled the criteria for uniquely mappable sequences (Section 2). Three umMRCs per integration site were generated (Supplementary Table S2).

**Figure 3:**
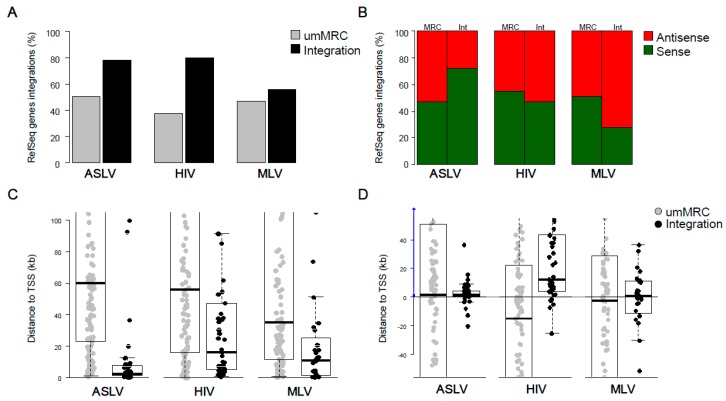
Analysis of genomic features at the integration sites of stable active proviruses. (**A**) Percentage of proviruses identified inside RefSeq Genes. (**B**) Orientation of proviruses inside RefSeq Genes relative to the transcription of targeted genes. (**C**) Distance of proviruses to the nearest TSS of RefSeq Genes (in kb). (**D**) Distribution of proviruses around TSS. The positive values mark the distance downstream from RefSeq Gene TSS. umMRC, uniquely mapped matched random control.

First, we analysed the frequency of integration into transcription units (TU, classified as genes in the RefSeq database) and the orientation of proviruses relative to transcription of targeted RefSeq Genes (Figure 3A,B). ASLV and HIV proviruses with stable expression were found to have the same frequency of approximately 80% in RefSeq Genes, which means a significant increase compared to umMRCs. On the other hand, the frequency of MLV stably expressed proviruses found in RefSeq Genes was lower than that of ASLV and HIV and were comparable to respective umMRCs. Interestingly, the orientation of proviruses relative to targeted RefSeq Genes showed different patterns for different vectors. ASLV stably expressed proviruses showed a preponderance of proviruses with sense orientations to RefSeq Genes transcription (*p* = 0.0225, Fisher’s Exact Test for Count Data). HIV proviruses displayed an equal proportion of proviruses in sense or antisense orientations to endogenous transcription. A striking majority of MLV proviruses were found in antisense orientation compared to that of targeted RefSeq Genes (*p* = 0.0489, Fisher’s Exact Test for Count Data).

Next, we measured the distance of stably expressed proviruses from the transcriptional start sites (TSS) of the RefSeq Genes (Figure 3C). All groups of proviruses accumulated significantly closer to TSS in comparison to respective umMRCs with medians of distances well below 20 kb. We also observed a similar accumulation close to CpG islands for ASLV and MLV and, to a lesser extent, for HIV (Supplementary Figure S2). We also checked the distribution of proviruses around TSS (Figure 3D). While ASLV and MLV proviruses distribution tended to centre in close proximity of the TSS, HIV proviruses distribution centred at a distance of 12 kb from TSS inside the gene body.

In conclusion, we have shown the differential distributions of stably expressed proviruses of ASLV, HIV and MLV. ASLV proviruses accumulated inside RefSeq Genes close to TSS and mostly in sense orientation to the gene transcription while proviruses of HIV also accumulated inside RefSeq Genes but in longer distances to TSS and in both orientations. There is no overrepresentation of stably active MLV proviruses inside RefSeq Genes but they were mainly distributed around the TSS mostly in the anti-sense orientation.

### 3.3. Stably Active Proviruses Associate with Active Genes

**Figure 4:**
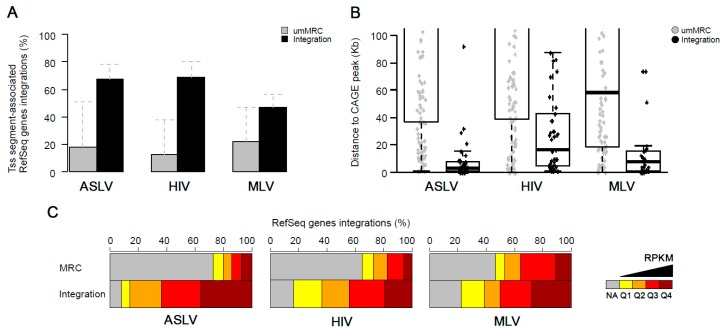
Transcriptionally active genes targeted by proviral integrations. (**A**) Percentage of proviruses identified inside RefSeq Genes associated with Tss chromatin state marking active TSS. Dashed whiskers show the original percentages of proviruses inside RefSeq Genes without selection for the presence of the Tss chromatin state. (**B**) Distance of proviruses to cap analysis of gene expression (CAGE) peaks that mark the site of active TSS. (**C**) Transcriptional activity of genes with proviruses. Genes were classified into 5 groups according to their mean read per kilobase per million (RPKM) mapped reads. NA marks group with no or very low transcriptional activity. Q1 to Q4 groups contain the genes with RPKM ≥ 1, where Q1 group represents the lowest quartile and Q4 the highest quartile.

As we have shown previously [39,40], the environment that is permissive for provirus expression can be correlated with the activity of targeted TUs. Therefore, we used a subset of RefSeq Genes, whose TSS associated with features defining active promoters, namely trimethylation of lysine 4 at histone 3 (H3K4me3), a Tss chromatin segment defined by ChromHMM [48] or peaks of transcription defined by CAGE [49]. Approximately 20% of umMRCs targeted these active RefSeq Genes whereas the frequencies of proviral integrations were approximately the same for all RefSeq Genes as well as the subset of active RefSeq Genes (Figure 3A, Figure 4A and Figure S3). The targeting of Active RefSeq Genes was significantly higher than targeting of respective umMRCs not only for ASLV and HIV but also MLV (*p* = 0.0148, Fisher’s Test for Count Data, Figure 4A), which was not different in targeting the all RefSeq Genes and respective umMRCS (Figure 3A).

We observed the same effect at the level of distance from the TSS of active RefSeq Genes (Figure 4B). Hits of umMRCs were distant to the TSSs of active RefSeq Genes in comparison to TSSs of all RefSeq Genes, whereas proviral integration sites were found to be of similar distances to TSSs of active RefSeq Genes as well as TSSs of all RefSeq Genes.

In order to address the activity of TUs targeted by integrations, we used publicly available RNA-seq data as described previously [40]. Briefly, TUs were divided into activity groups according to their mean read per kilobase per million (RPKM) mapped reads of RNA-seq datasets. In correspondence to the previous selection for active RefSeq Genes, RNA-seq data showed that the targeted TUs mostly exhibited transcriptional activity with a RPKM ≥ 1 (Figure 4C).

These results demonstrated that the proviruses selected for stable transcriptional activity accumulated near the TSSs of active TUs, likely because of their active transcription-associated chromatin environment, which is also permissive for provirus expression.

### 3.4. Stably Active Proviruses Associate with Active Chromatin and Active Regulatory Segments

**Figure 5:**
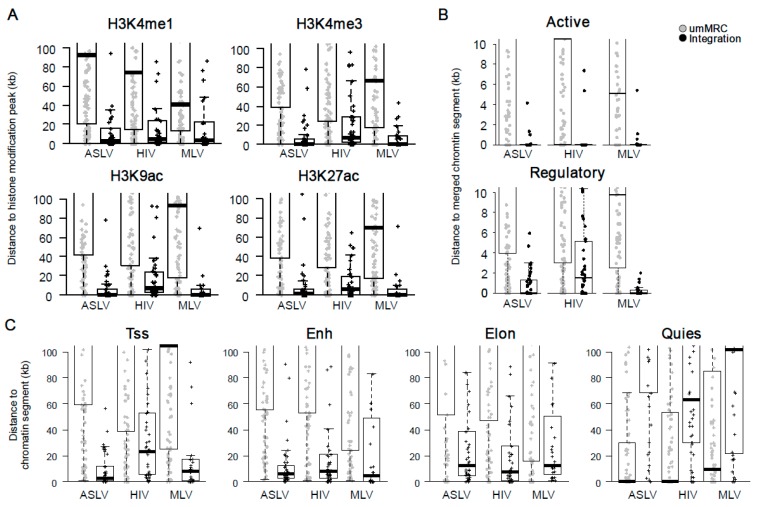
Epigenetic features at the integration sites of stably expressed ASLV, HIV and MLV proviruses. (**A**) Distances of proviruses to the peaks of selected histone modifications that are associated with active promoters and enhancers. (**B**) Distances of proviruses to merged chromatin segments. Active segments are chromatin segments which are associated with transcriptionally active chromatin. Regulatory segments are chromatin segments associated with active promoters and enhancers. (**C**) Distances of proviruses to 4 of the 25 chromatin segments analysed, Tss, transcriptional start site segment; Enh, enhancer segment, Elon, elongation segment and Quies, polycomb-repressed segment. The distance is measured as absolute distance to the nearest peak of a histone modification or a chromatin segment. Each dot represents a single provirus. Black dots, real integrations; grey dots, umMRCs.

Epigenetic features of the chromatin environment at the site of integration were first described by comparing the distances of the proviruses and umMRC to peaks of eleven histone modifications (Supplementary Figure S4A) defined for the K562 cell line by the ENCODE project. ASLV, HIV and MLV proviruses with stable transcriptional activity accumulated in short distances to peaks of epigenetic modifications that are associated with active chromatin but not with markers of heterochromatin. A common feature of all groups of retroviral vectors was the short distance to the peaks of mono- and tri-methylated H3K4 and acetylated H3K9 and H3K27 histones (Figure 5A). HIV proviruses displayed a more relaxed pattern with the mean distance to the peaks of these four histone modifications that was further way when compared to ASLV and MLV.

We then calculated the distances of the long-term active proviruses to the chromatin segments, which are defined by the combination of genomic and epigenomic features that are specific for distinct functional parts of genome specified for the K562 cell line [47]. First, we merged functionally related chromatin segments creating broader segments of Active chromatin considering transcribed parts of genome together with active promoters and enhancers and the Regulatory chromatin considering active promoters and enhancers (see Section 2.9 and [40]). The stably active proviruses of ASLV, HIV and MLV showed a close association with active chromatin (Figure 5B top). In fact, the most stably active proviruses were found inside some of the 16 segments associated with Active chromatin. Stably active proviruses were also found in close proximity to the merged Regulatory segments, where the highest median distance (ca 1.5 kb) was observed for HIV proviruses (Figure 5B bottom). We then calculated the distances from all 25 chromatin states available (Supplementary Figure S4B). In agreement with previous results, the proviruses of all groups analysed were found close to the chromatin states associated with active TSSs. Even though stably active HIV proviruses showed significantly longer distances to TSS state than ASLV and MLV, these differences were lost when distances to chromatin states flanking active TSS (TssF, PromF) were analysed. More interestingly, the stably active proviruses of ASLV, HIV and MLV were found in close proximities to enhancer-associated chromatin states.

Together, the analysis of epigenomic and functional landscape of stably active proviruses showed that regardless of origin of provirus, stable expression of provirus associates within the proximity to the genomic loci driving genomic transcription. Although stably active proviruses of ASLV and MLV were found closer to the features associated with an active TSS than HIV proviruses, the proviruses of all three groups were found to harbour enhancer-proximal loci.

### 3.5. Enhancer Proximity Is Not a Function of Active Gene Targeting

As shown in Figure 5B, almost all the stably active proviruses of ASLV, MLV and HIV were found inside or very close to active chromatin segments, active TSS and enhancers. Furthermore, the majority of stably active ASLV and HIV proviruses were found in genes. Therefore, we sought to investigate if the proximity to active TSS and enhancers can be reached by preferential targeting of active genes. For this purpose, another level of matching for umMRC was added by selecting umMRCs that show a similar distance to the active RefSeq Genes as integration sites of stably active proviruses. A new group of matched random controls was then called active gene-matched umMRC (agMRC, see Section 2 and Supplementary Table S2). The targeting of active RefSeq Genes by real integrations and agMRCs is shown in Supplementary Figure S5A and the distances to TSS of active RefSeq Genes are found in Supplementary Figure S5B.

**Figure 6:**
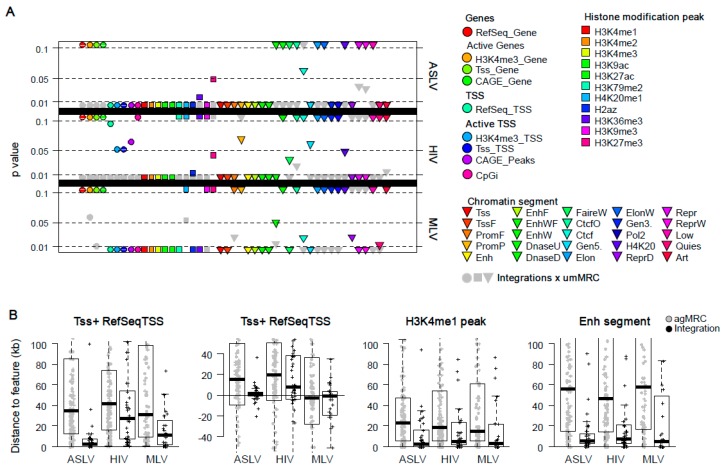
Comparison of integration sites of stable active proviruses and respective active gene-matched random controls. (**A**) Statistical *p*-values of differences between agMRC and integration sites are represented by coloured circles (genes), squares (peaks of histone modifications) and triangles (chromatin segments) and aligned with x axis. agMRCs are created to match the proviral integration sites with the frequency of targeting RefSeq Genes with active TSS part according to the chromatin segment classification. Thin dashed lines mark the *p*-values of 0.01, 0.05 and 0.1. Values outside the range from 0.01 to 0.1 are located at the lower/upper edge of the chart beyond the dashed lines. Grey symbols represent the *p*-values of testing integration sites against umMRC. (**B**) Examples of the charts representing the values of agMRCs and proviral integration sites. From left to right: Tss^+^RefSeqTSS, absolute distance; Tss^+^RefSeqTSS, distribution around TSS; peaks of H3K4me1 enrichment; enhancer segments.

Figure 6A displays the statistical significance of the differences in targeting or distance to genomic or epigenomic features between real integrations and umMRC. Figure 6B exemplifies the distances of agMRC and real integrations to selected features (for the full overview, see Supplementary Figure S6A,B). As a result of matching, the data showed no significant difference of active RefSeq Genes targeting between stably active proviruses and agMRCs. For ASLV, the distances to most histone modifications differed significantly between agMRC and stably expressed proviruses, while differences in distances to some active chromatin segments were insignificant. Most importantly, the stably expressed proviruses of ASLV were significantly associated with active TSS and strong enhancers. For the HIV and MLV dataset, the trend depicting the loss of significant differences between stably active proviruses and agMRC in the set of active chromatin segments and the preservation of the significant difference for active TSS and enhancers was notable. Interestingly, the data for HIV showed the stably active proviruses were significantly associated to active TSS and enhancers as well as to the histone marks characteristic for those regulatory segments—H3K4 methylation and histone acetylation.

Together, these data show that when real integration sites are compared to their matched controls that mimic the active gene targeting of proviruses, the close proximity to the features that are characteristic for active TSS and enhancers are preserved as a hallmark of stably active proviruses of ASLV, HIV and MLV.

## 4. Discussion

The heterogeneity of proviral expression and the role of chromatin environment in proviral transcriptional activity, for example in HIV infection in vitro and in vivo, are still unclear as some controversies remain to be resolved (reviewed in [50,51]). In our study, we investigated the transcriptional stability of distinct retroviral vectors derived from ASLV, HIV and MLV and the association of long-term active proviruses with the functional genomic and epigenomic features at the site of integration.

In our previous studies, we showed that most ASLV proviruses in human cells are effectively silenced and that rare transcriptionally stable proviruses strongly depend on their position in the host genome [39,40]. The long-term stable ASLV proviruses are preferentially found in close proximity downstream to active TSS. The same vectors equipped with CpG-island core sequences are less dependent on TSS-proximity but stably active proviruses are found close to the sites epigenetically characterized as active enhancers. In this study, we compared ASLV [40] with newly analysed stably active HIV and MLV proviruses.

Unlike ASLV, the majority of HIV and MLV proviruses transcriptionally active early after infection were transcriptionally stable during the subsequent culturing. This confirmed the previous observation of cellular clones that were productively infected with HIV-derived vector, which kept the provirus expression stable for months after infection [52]. Our system which was based on the fluorescent reporter demonstrated that most proviruses active 3 dpi maintained their transcriptional activity for at least 30 dpi. For MLV, the majority of proviruses that were selected for reporter activity were transcriptionally stable up to 60 dpi. In contrast to ASLV, HIV and MLV are mammalian retroviruses whose LTRs contain enhancer sequences that resemble CpG-islands with Sp1 binding sites. Both HIV and MLV also preferentially integrate into specific functional regions: HIV prefers gene bodies of active TUs, whereas MLV preferentially integrates close to active TSS and enhancers. Either promoter properties or preferential integration into the transcription-permissive environment may play role in HIV and MLV stable expression and the effect of both factors should be addressed in future studies.

An important issue of the splinkerette PCR is the comparison with random matched control, which normalizes the imperfect coverage of insertion sites and potentially biased amplification in multiplex PCR. Our datasets of integration sites were compared to random controls matched on several levels—the distance to restriction enzyme recognition sites, the mappability of the region and the distance to specific chromatin segment. Only significant differences compared to the random matched controls were used to draw conclusions.

We found similar frequencies of stably active ASLV and HIV proviruses integrated in active RefSeq Genes. In the case of ASLV, their accumulation in active RefSeq genes results from the selection eliminating the silenced proviruses [40]. HIV proviruses are naturally targeted to these regions by the integration machinery and, correspondingly, most proviruses occur in the expression-permissive regions at the time of infection [2,3,4,5]. Unlike stably active ASLV proviruses, we found the selected HIV proviruses farther from the active TSS but in close proximity to the enhancers. We observed previously the similarly high frequency of selected proviruses in active genes and close to enhancers for the ASLV vector modified by CpG island core sequence [40]. The preference of enhancers was previously described as a hallmark of natural MLV [7,53] but not HIV integration. However, it was recently reported that active HIV proviruses compared to their silenced counterparts are closer to H3K27 acetylation-enriched regions, i.e. markers of active TSSs and enhancers. The proviruses which are silenced but can be reactivated by histone deacetylase inhibitors are found closer to H3K27 acetylation-enriched sites than the silent proviruses that do not respond to this treatment [34]. Also, the targeting of active TUs facilitated by LEDGF/p75 seems to be important for the transcriptional activity of HIV proviruses since the disruption of LEDGF/p75-integrase interaction by LEDGINs increases the proportion of latent reactivation-resistant reservoir in the HIV proviral population [33].

Stably active proviruses of MLV did not accumulate in active RefSeq Genes but were concentrated around TSSs, with the median distance comparable to that observed in ASLV and with even shorter distances around enhancers. While ASLV accumulated close to active TSSs and enhancers only after long-term selection for proviral expression, MLV preferentially targeted active TSS and enhancers due to the interaction of integrase with BET proteins [16,17,18]. Thus, we cannot assess the contribution of selection to the distribution of MLV proviruses observed here. This could be studied using the BET-independent gammaretroviral vectors that integrate outside the active TSS and enhancers [20,21]. In this context, a recent study [22] reported that the next-generation BET-independent MLV-derived vectors showed the same stability of expression without genotoxicity when compared to a wild-type MLV-derived vector.

The weak preference of ASLV integration for active genes and close-to-random distribution of non-selected ASLV proviruses is advantageous for insertional tumorigenesis studies. Malhotra et al. [54] compared the integration pattern of ALVs in cultured cells and B-cell lymphomas, the most common malignancies caused by ALV. In accordance with the clonal hypothesis, authors observed strong selection for proximity to TSS during the progression and even metastasis of tumours. *TERT* and *Myb* genes were found among the most clonally expanded integrations showing the link between the provirus accumulation and common integration sites widely studied using ALV [55,56]. We also previously studied the distribution of acutely transforming ASLV proviruses in solid tumours that grow due to the provirus/oncogene expression. We observed here accumulation of proviruses in genes transcriptionally active in a broad range of tissues and avoiding of genes transcribed in a tissue-specific manner [57].

**Figure 7:**
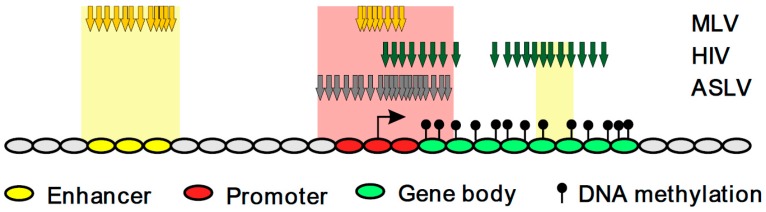
The model recapitulating the distribution of stably active proviruses with regard to the genomic and epigenomic features of the integration sites. Yellow arrows, MLV; green, HIV, blue, ASLV.

Based on our comparison of stably active ASLV, HIV and MLV proviruses, we suggest that the proximity to an active TSS is important for the stable expression of proviruses with silencing-prone promoters such as ASLV and HIV, whereas the proximity to enhancers favours the stable activity of proviruses with silencing-resistant promoters such as MLV or our previously described ASLV vector artificially equipped with a CpG island core sequence [40]. Interestingly, transcriptionally active HIV proviruses located near enhancers were found inside active TUs similarly as was observed with CpG-island-modified ASLV [40]. Therefore, we can hypothesize that the TU bodies, the most densely CpG-methylated genomic regions [58,59], represent a transcription-permissive environment for proviruses that are resistant to DNA methylation, e.g. due to the presence of a CpG island within their LTRs (see the model in Figure 7).

Although our data points to the importance of chromatin content in proviral transcriptional activity, further studies that include the analysis of a larger set of integration sites together with single-provirus expression profiles will be needed to confirm the proposed model. Our clonal approach used here and in previous studies offers a longitudinal record of single provirus expression and correlation with the properties of respective integration site. The drawback of this method, the labour intensity and low throughput, has recently been overcome by Chen et al. [34], who coupled the single provirus approach with high-throughput methods. Also, the retargeting strategies that are now available could be used to solve the question of whether the integration site plays an important role in the stability of provirus expression.

The results of this study are important particularly for the design of retroviral vectors. Our data shows that proviruses selected for their long-term expression stability accumulate at transcriptionally active chromatin in the vicinity of regulatory elements such as promoters and enhancers. This raises the risk of genotoxicity and clonal expansion, which should be taken into account and addressed in future studies.

## Supplementary Materials

The following are available online at http://www.mdpi.com/1999-4915/10/3/116/s1. Figure S1: Relative GFP fluorescence intensity in cell clones transduced with ASLV, HIV and MLV reporter proviruses and expanded 30 dpi. The relative GFP fluorescence is calculated as median intensity of GFP^+^ cells/minimal median intensity of GFP- clones of the same group. All clones of a given provirus (black dots) are compared with clones selected as stable during the subsequent culture (green dots); Figure S2: Distances of proviral integration sites to the nearest CpG island. Each dot represents a single provirus. Black dots, real integrations; grey dots, umMRCs; Figure S3: Frequency of proviruses integrated in the RefSeq Genes. Percentage of proviral integration sites and umMRCs in all RefSeq Genes (All) or RefSeq Genes filtered by the overlap of RefSeq Genes TSS with markers of active TSS. Grey dashed lines mark the level of integrations into all RefSeq Genes. agMRC, active gene-matched random controls; Tss, TSS chromatin segment; H3K4me3, trimethylation of lysine 4 of histone 3; CAGE, cap analysis of gene expression peaks; Figure S4: A comprehensive overview of epigenetic features at the integration sites of stably expressed ASLV, HIV and MLV proviruses. (**A**) Distance of proviral integration sites and umMRCs to histone modification peaks. (**B**) Distance of proviral integration sites and umMRCs to chromatin segments; Figure S5: Targeting of active genes by proviral integrations and agMRCs. (**A**) Percentage of proviral integrations and agMRCs in all RefSeq Genes (All) or RefSeq Genes filtered by the overlap of RefSeq Genes TSS with markers of active TSS (Tss^+^, H3K4me3^+^ and CAGE^+^). (**B**) Distance to TSS of all RefSeq Genes or RefSeq Genes filtered by the overlap of RefSeq Genes TSS with marker of active TSS as in A. agMRC, active gene-matched random controls; Tss, TSS chromatin segment; H3K4me3, trimethylation of lysine 4 of histone 3; CAGE, cap analysis of gene expression peaks; Figure S6: Distance of integration sites and agMRCs to the peaks of histone modifications and chromatin segments. (**A**) Distance of proviral integration sites and umMRCs to histone modification peaks. (**B**) Distance of proviral integration sites and umMRCs to chromatin segments. agMRC, active gene-matched random controls; Table S1: The list and characterization of retrovirus integration sites analysed in this study. Retroviral vector (ASLV- HIV-, or MLV-derived), the serial number of the cell clone, targeted chromosome, integration position and orientation and restriction enzyme used for splinkerette PCR are given in the table; Table S2: The list and characterization of Matched Random Controls created for comparison with real integrations analysed in this study. The respective vector (ASLV-, HIV-, or MLV-derived), MRC type (either uniquely mapped matched random controls or active gene matched random controls), serial number of the integration site, targeted chromosome, genomic position and transcriptional orientation are given in the table.
