# Supplementary figures and images for: Proviruses with Long-Term Stable Expression Accumulate in Transcriptionally Active Chromatin Close to the Gene Regulatory Elements: Comparison of ASLV-, HIV- and MLV-Derived Vectors
Source: Viruses. 2018 Mar 8;10(3):116. doi: 10.3390/v10030116 (PMC5869509; doi:10.3390/v10030116)

# Relative GFP fluorescence intensity of the clones at 30 dpi

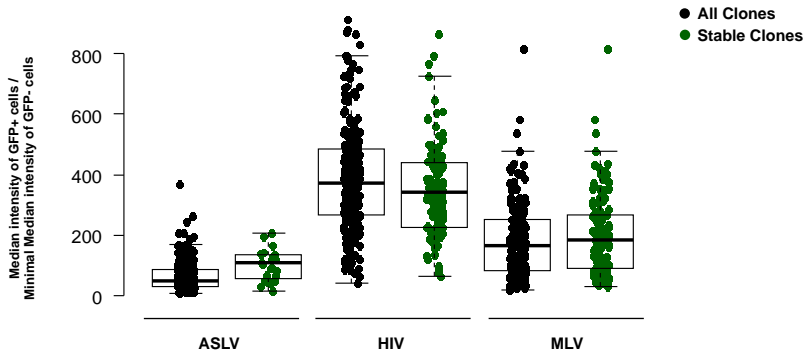

Supplement: Supplementary file 1 [file viruses-10-00116-s001.zip › Supplementary Figure 1.pdf]

# CpG island

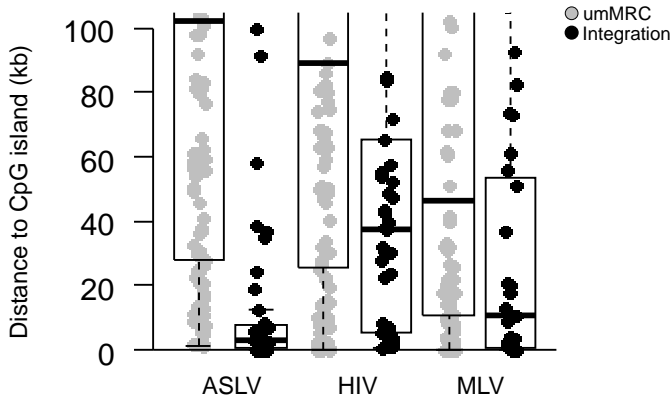

Supplement: Supplementary file 1 [file viruses-10-00116-s001.zip › Supplementary Figure 2.pdf]

**All**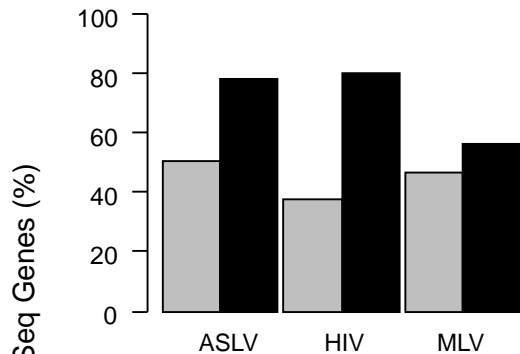**Tss+**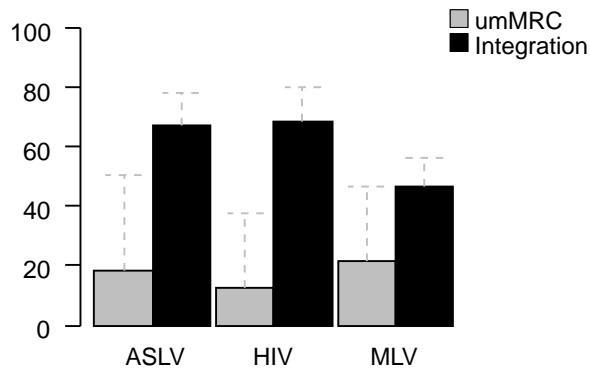**H3K4me3+**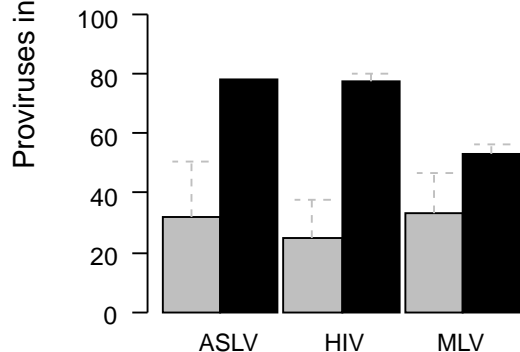**CAGE+**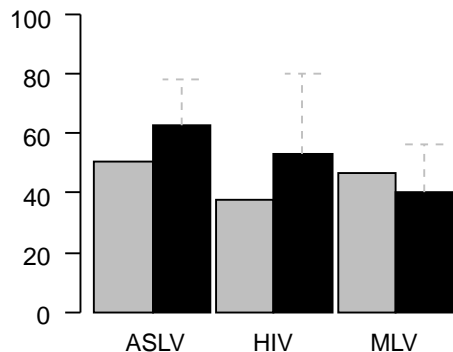

Supplement: Supplementary file 1 [file viruses-10-00116-s001.zip › Supplementary Figure 3.pdf]

**A**

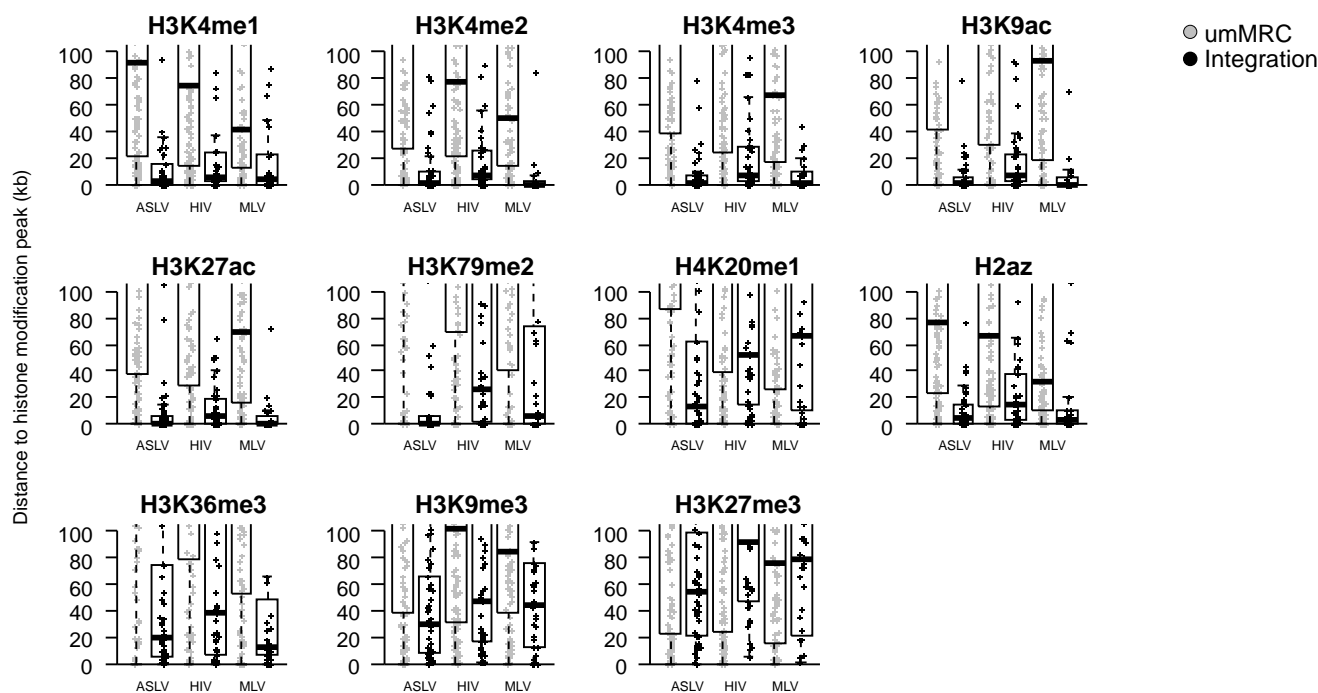

● umMRC  
● Integration

**B**

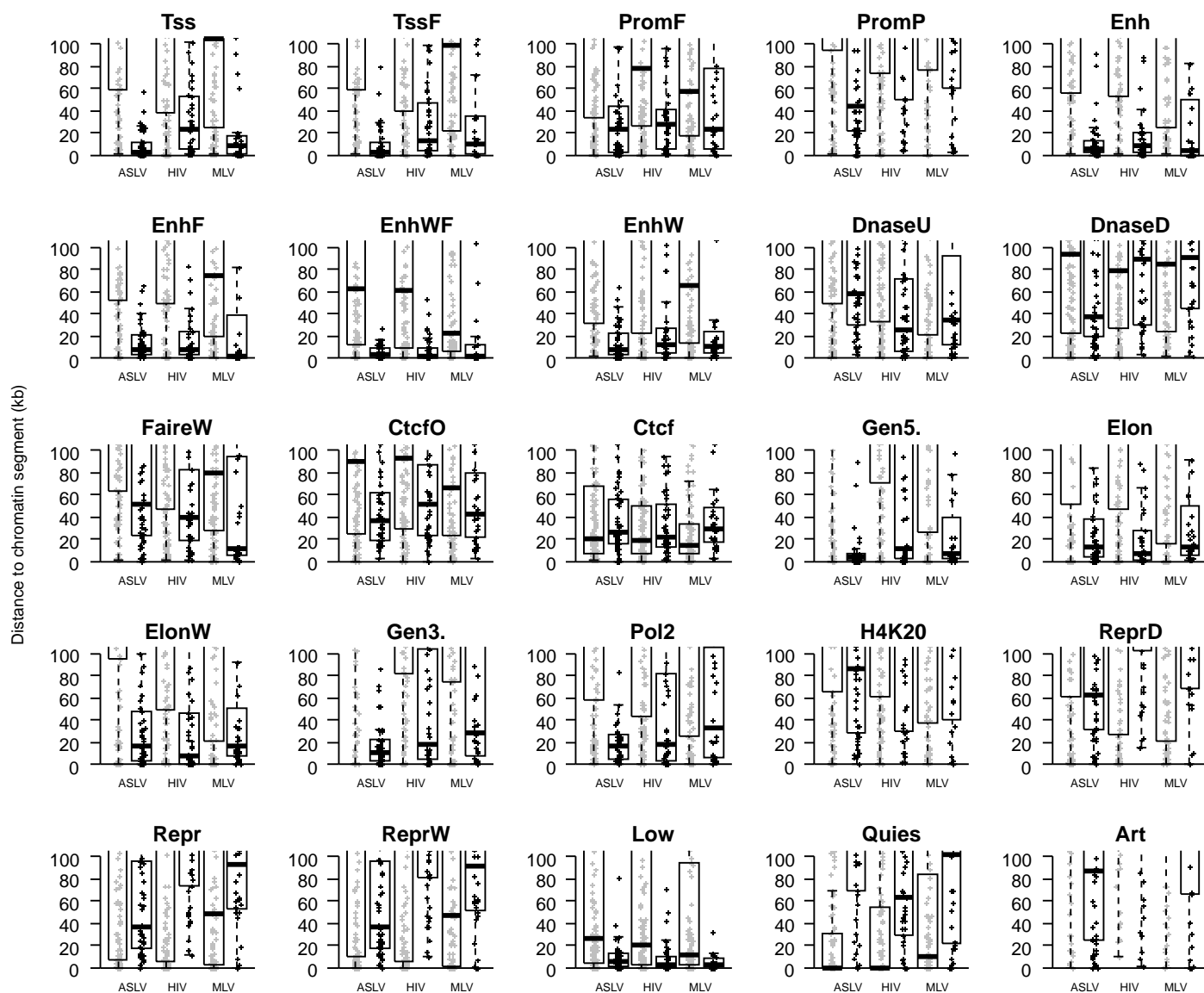

Supplement: Supplementary file 1 [file viruses-10-00116-s001.zip › Supplementary Figure 4.pdf]

**A**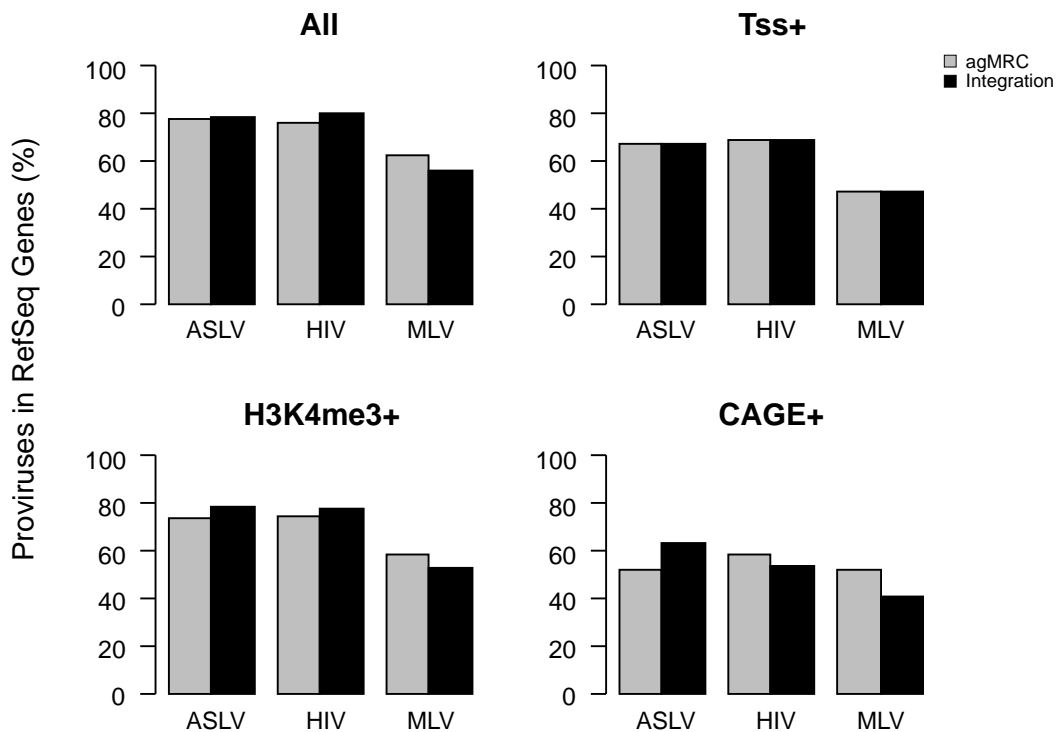**B**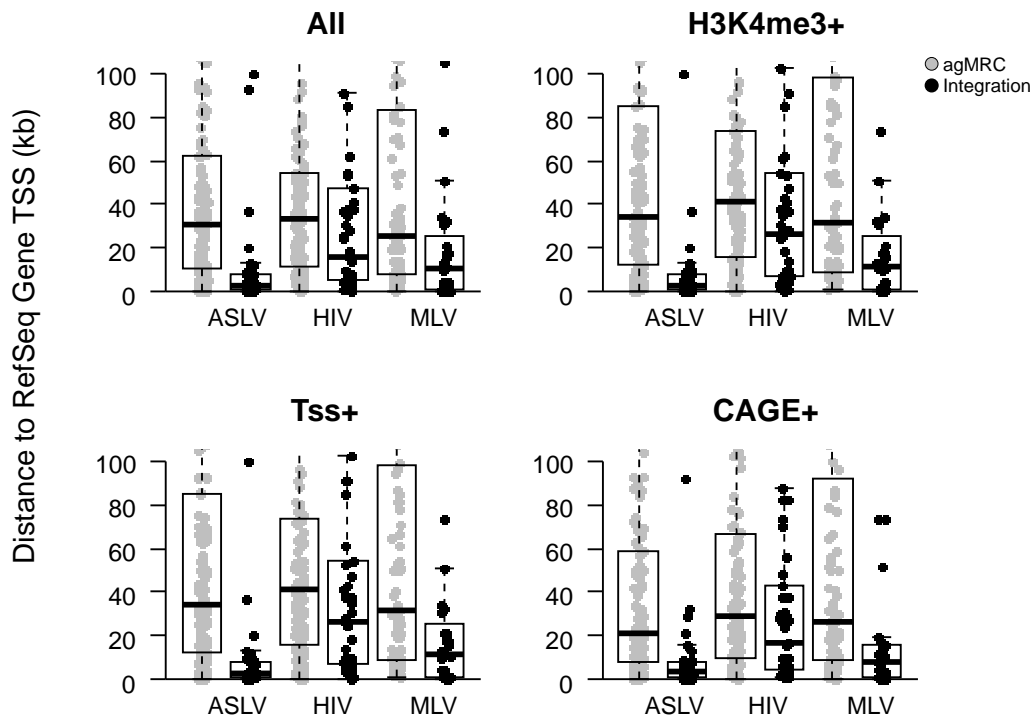

Supplement: Supplementary file 1 [file viruses-10-00116-s001.zip › Supplementary Figure 5.pdf]

**A**

Distance to histone modification peak (kb)

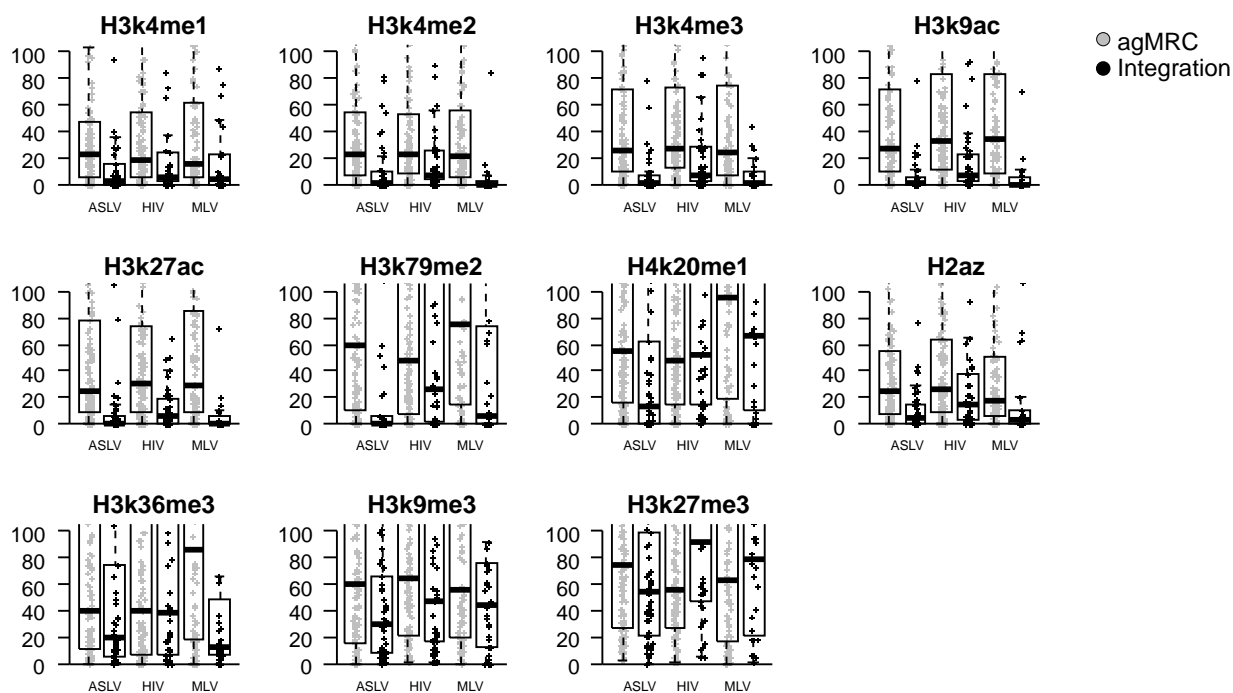

**B**

Distance to chromatin segment (kb)

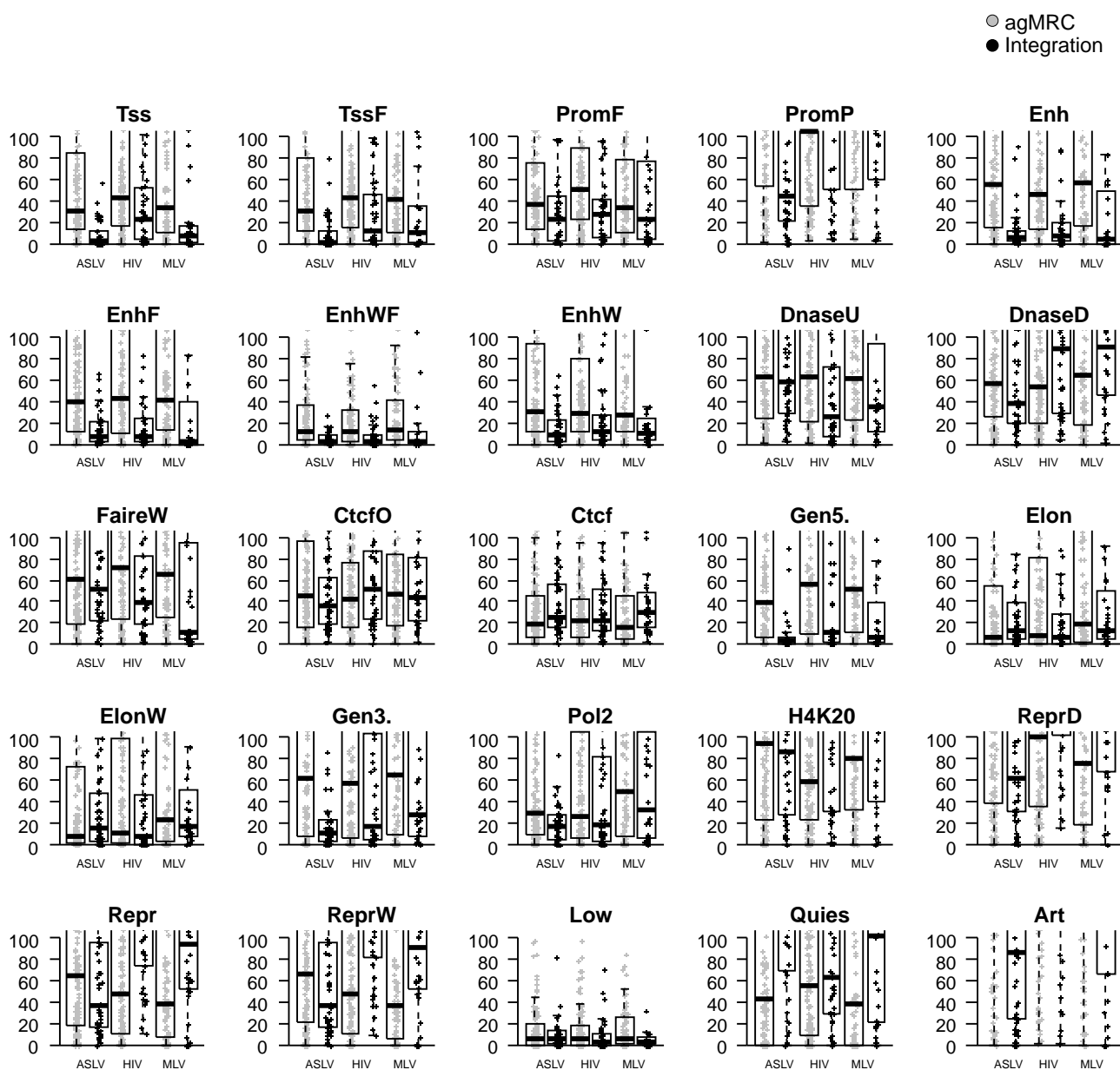

Supplement: Supplementary file 1 [file viruses-10-00116-s001.zip › Supplementary Figure 6.pdf]
